# Book Reviews

**Published:** 1889-09

**Authors:** 


### Book Reviews.

A Clinical Atlas of Venereal and Skin Diseases, including Diagnosis, Prognosis and Treatment, by Robert W. Taylor, A. M., M. D., surgeon to Charity Hospital, New York, and to the department of venereal and skin diseases of the New York Hospital; late president of the American Dermatological Association. Illustrated with one hundred and ninety-two figures, many of them life-size, on fifty-eight beautifully colored plates; also many large and carefully executed engravings through the text. Lea Brothers & Co., Philadelphia, 1888.

We are in receipt of the first six parts of this excellent work, which does much credit to both author and publishers. The paper, binding, type, general arrangement of plates, etc., are all that could be desired.

The distribution of the plates throughout the work, so that each colored plate immediately follows the descriptive text, is a commendable feature.

Dr. Taylor's style is too well known to our readers to need comment. His terse, forcible manner of expressing himself is especially admirable in a work of this character. He presents clearly, without ostentation, the clinical facts which have come under his observation. The first three parts are devoted to venereal diseases, beginning with gonorrhœa.

Dr. Taylor does not believe in the specificity of gonorrhœa, nor does he accept the theory now generally held by more advanced pathologists in genito-urinary diseases, that the gonococcus of Neisser is the cause of gonorrhœa, and while he does not consider its presence merely a coincidence, he is unwilling to accept it as a constant concomitant, and does not think the therapeutics of gonorrhœa should be at all modified on account of its existence in the discharges. He adheres to the generally accepted plan of treatment, and gives many excellent prescriptions for injections, and internal administration, though he presents nothing new.

Of chancroid, little need be said. Dr. T. does not consider it a specific disease. The colored plates showing the different stages and situations of the ulcers are excellent.

The characteristic differences between herpes, chancroid and chancre are clearly delineated. Syphilis is next in order, and the various syphilides with their characteristic appearances, are accurately shown up by a series of excellent plates, clearly outlining the eruptions in their protean types. The descriptive text is clear, concise, entertaining. The author is a firm believer in the efficacy of a long continued course of mercurials, and gives a number of formulæ for the combinations which have proven most valuable in his hands.

Parts IV, V and VI are devoted to the consideration of skin diseases proper, beginning with general observations. The various lesions are next taken up and described systematically; then comes classification, that of the American Dermatological Association being adopted.

The different diseases are next taken up, beginning with erythema multiforme ; next e. nodosum. Then come eczema, psoriasis and favus, concluding Part IV.

Part V considers in order named pediculosis, erythema faciei and ephemeral erythema, erythema circinatum, herpes iris and erythema serpens, tinea versicolor, tinea tonsurans, pityriasis rubra, dermatitis exfoliativa, impetigo herpetiformis.

Part VI includes urticaria, pemphigus, tinea, tricophytina barbæ, tinea circinata, ecthema, laupus, erythmatosus, and herpes zoster.

Well executed plates and concise practical descriptive text accompany each subject, making the work entertaining in every detail.

Next to actual observation of the diseases themselves, come clinical atlases in aiding us in arriving at correct diagnosis without which our treatment must be empirical and generally futile.

Dr. Taylor has done the profession much service in giving to it this excellent work. F. W. M.

